# The persistence of history: Racism, anti-Blackness, and the causes of mental ill health, *c*.1800–2020

**DOI:** 10.1177/09526951251331378

**Published:** 2025-06-20

**Authors:** Rebecca Wynter, Niyah Campbell, Sarah Chaney, Sarah Marks

**Affiliations:** University of Amsterdam, The Netherlands; University of Birmingham, UK; 1724 University of Birmingham, UK; Queen Mary University of London, UK; Birkbeck, University of London, UK

**Keywords:** anti-racism, historiography, mental health, race, transcultural psychiatry

## Abstract

This article offers an overview, historicising how both ‘race’ and racism have been seen as an aetiological root for mental ill health in people racialised as black, and contextualising the rise of transcultural psychiatry. Heeding the calls of the few historians to study race, mental health, and transcultural psychiatry in the metropole and across the globe, this article includes a wider variety of voices in order to create a dialogue between these histories. Drawing extensively on the global historiography and embracing contemporary work that demonstrates the persistence of racism in psychiatry and research, we use medical publications and a prosopographical lens to explore the push from radical psychiatrists to uproot racism and anti-b/Blackness in psychiatry and to recognise the impact of racism on mental health. This article considers the practice and activism of three late 20th-century UK-trained clinicians: Jamaican Frederick Hickling (1944–2020), British Jamaican Aggrey Burke (b. 1943) and British Sri Lankan Suman Fernando (b. 1932). By shifting the gaze of the historiography, we argue that beyond the towering figures of Frantz Fanon and Thomas Adeoye Lambo, psychiatrists were engaged in anti-racism and decolonisation using history—and doing so long before decolonisation became a watchword for historians globally.

Content Warning: The following overview contains words and explores views that are now rightly considered offensive. These expressions are used only in their original context, and then only to generate an idea of how such attitudes have informed aetiology and mental health science, and how this thinking survives through intellectual baggage and the continued use of concepts formed in the past.

According to the latest NHS figures, Black men in the UK aged between thirty-five and forty-nine are four times more likely than white men to be detained under the Mental Health Act and ten times more likely to be under a Community Treatment Order.… The figures for Black women are also disproportionate: roughly six times more than white women. Consider for a moment how relatively rare incidences of serious mental ill health are in Black men and women of a similar age living in the Caribbean. I don’t need anyone to explain that to me. Living in the UK … Black pain is denied. (Harewood, 2021: 195–6)

As a young, Birmingham-born man emerging from the Royal Academy of Dramatic Art (RADA), David Harewood did not expect to find himself detained under the 1980 Mental Health Act on a psychiatric ward, after experiencing an episode of psychosis. Thirty years later, after establishing a successful career, Harewood reflected on his experiences in a BBC documentary, *Psychosis and Me* (2019), and a 2021 book. In both, Harewood critically examined the commonplace notion that rates of psychosis among ‘the Black population’ are higher solely through the systemic racism of the police and mental health services. Against this notion, he drew instead on his own life and the work of contemporary mental health experts to argue that the experience of racial discrimination should itself be understood as a primary causal factor of psychosis.

This article offers an overview of the past two centuries, ending in 2020, the year George Floyd was killed by US police. It historicises how both race and racism have been seen as an aetiological root for mental ill health. At its heart is an exposition of the role of culture in how mental health and illness are conceptualised. German Berrios and Ivana Marková have argued that mental illnesses undergo ‘refurbishments[, obtaining] their meaning from the historical periods in which they are allowed to be active’ (2017: 115–16). However, we see ideas and diagnostic terms as freighted with their history *despite* their culturally specific meaning at the point at which they are deployed: the past persists.

This article also considers the practice and activism of three late 20th-century UK-trained clinicians who used culture to understand how prejudice has shaped experiences and mental health: Jamaican, Frederick Hickling (1944–2020), British Jamaican, Aggrey Burke (b. 1943), and British Sri Lankan, Suman Fernando (b. 1932). The following pages will: contextualise the emergence of transcultural psychiatry; indicate that figures like Hickling, Burke, and Fernando, though often sidelined, altered thinking in Jamaica and England; and demonstrate that, beyond the towering figures of Frantz Fanon and Thomas Adeoye Lambo, psychiatrists were engaged in anti-racism and decolonisation using history—and doing so long before decolonisation became a watchword for historians globally, and pointedly in ‘Western’ countries. As Ana Antić has argued, ‘Histories of transcultural psychiatry ought to include a much broader variety of voices than they have done so far’ (Antić, 2022: 23). Martin Summers has stated the need to ‘put our histories of race and psychiatry into conversation with the histories and historiography of colonial psychiatry’ (Summers, 2010: 61); this article aims to do just that. Drawing extensively on the global historiography, we use medical publications and a prosopographical lens to explore the push from radical psychiatrists to both uproot racism in psychiatry and recognise the impact of racism on mental health.

The article will therefore be split into four main sections, largely in chronological order, beginning with key themes that have dominated psychological and psychiatric discussions around race since the 1700s. While the deep roots of this causation go back further, the first section introduces how concepts of ‘civilisation’ (coded as white), ‘primitivity’ (as b/Black), and migration (‘other’)—birthed in so-called ‘Western’ thinking—became explanations for resilience and vulnerability to mental health issues. Manifestations and experiences of racism vary across space and time. However, this article will suggest that in the praxis and organisation of contemporary mental health research, the lineage of racist ideas and anti-b/Blackness can be traced from the 1700s to the present day within the UK and US and globally.

The second section will consider the early 20th century, embracing the expansion of Black intellectual space and the challenge to racialised models of mental illness rooted in statistics. Section three discusses the impact of post-1945 shifts—the global desire for peace and understanding, the collapse of empire, new patterns of immigration, and radical, anti-racist thinking, in part through transcultural psychiatry. We will chart key late 20th-century aetiological re-evaluations via the work of three transcultural psychiatrists. Frederick Hickling was a profoundly influential psychiatrist in Jamaica and innovator of ‘psychohistoriographic cultural therapy’. Aggrey Burke, the first Black NHS forensic psychiatry consultant, challenged racial stereotypes around suicide and helped expose racism in medical training. Suman Fernando, whose work on depression in Jewish communities was ‘the first MD thesis at Cambridge in the field of “transcultural psychiatry”’, later conceptualised psychiatry as systemically racist in his 1988 book *Race and Culture in Psychiatry* (Williams, 2012: 168).

The final section summarises mental health science around race and aetiology since the 1990s. Here, we highlight the shift towards individualisation and the recentring of biological science in causation, before the wider adoption of transcultural psychiatric sentiments in an expressly socio-cultural and anti-racist path for exploring and understanding aetiological origins. We also outline the ongoing exclusion of those racialised as ‘non-white’ in research to demonstrate the persistence of racism in the field of mental health.

## Civilisation, race and mental health (18th and 19th centuries)

Since the 18th century, one question has doggedly persisted: ‘Does civilization cause more mental illness than simpler stages of cultural development?’ (Rosen, 1959: 8). In *The English Malady* (1733), Scottish physician George Cheyne answered an emphatic yes. Just as the ancient Greek civilisation had ‘sunk into *Effeminacy*, *Luxury*, and *Diseases*’, so imperial Britain was likewise afflicted (Cheyne, 1733: 56–8). By taking new commodities from overseas, British civilisation and ‘nervous disorders’ boomed in tandem. Conversely, Cheyne argued, ‘primitive people had been so healthy as to have no need of medicine’ (Porter, 1991: xxviii).

This conceptualisation—that white-racialised Christian nations were ‘civilised’ and sick, and non-Christian societies, racialised as ‘non-white’, were ‘primitive’ and healthy—was deeply ingrained (Keller, 2001; Summers, 2010, 2019). Indeed, in 1866, over 130 years after Cheyne's claims, leading English psychiatrist Henry Maudsley took this for granted, citing ‘civilization’ as the first predisposing cause of mental health issues. ‘Travellers’, he continued, ‘are certainly agreed that [mental illness] is a rare disease amongst barbarous people.… In different civilized nations’, he continued, ‘there is … an average of about one insane person in five hundred inhabitants’ (Maudsley, 1966[1867]: 200–1).

The use of statistics to argue that ‘uncivilised’ people were protected from mental illness persisted well into the 20th century. As historian Matthew Heaton has shown, the rates of institutionalisation logged between 1936 and 1953 ‘led to a general belief that incidence of mental illness was lower among African populations than’ European, with England and Wales standing at 4 per 1000 people; South Africa at 1.2 per 1000; Gold Coast (Republic of Ghana) at 0.3; Kenya, 0.1; and Nyasaland (Malawi), 0.06 (Heaton, 2008: 89).

Certainly, European Empires were a vector for distributing ideas about ‘civilisation’, mental health and ‘primitive peoples’. Richard C. Keller argued that for the early 20th-century French, ‘reforming the Maghreb's institutions of madness represented a major accomplishment for the mission to “civilize” North Africa’ (Keller, 2007: 84). Psychiatry was central to the ‘civilising’ infrastructure imposed on colonised places, along with asylums and other institutions, including prisons and hospitals. Yet, as Len Smith and Jonathan Sadowsky point out, different colonising nations had different cultures of governance, and therefore oppression, and held influence beyond settler-colonised countries (Smith, 2014: 4; Sadowsky, 1999: 98). Nevertheless, European framings of how and why Africa did not already have ‘modern’ societies shaped how ‘the African mind’ came to be seen and, according to many Europeans, this in itself proved Africans’ intellectual inferiority and stunted evolution. This flattening of the culture and diversity of an entire continent encouraged colonial psychiatrists to explain ‘the African mind’ as a universal and distinct other (Heaton, 2008: 75–6). Despite refined thinking around civilisation/savagery (Thomson, 1999), even in the 1962 ninth edition of Henderson and Gillespie's profoundly influential *Textbook of Psychiatry*, the authors carried the baggage of the past, asserting that ‘there is evidence … that migration or other rapid and extensive social changes (for example, those due to the introduction of Western civilization to a primitive society) may be associated with an increased rate of mental breakdown’ (Henderson and Batchelor, 1962: 75).

These freighted terms, ‘civilisation’ and ‘primitive’, have been inseparably bound to Black migration, seen plainly in American discussions about African Americans inflected through increasingly violent pro-enslavement arguments in the lead up to the Civil War (1861–5). In 1849, for example, Samuel A. Cartwright led doctors tasked by Louisiana State Medical Convention to investigate conditions framed as ‘peculiar’ to those racialised as ‘negro’. Among a variety of different disorders, all with profoundly racist framings and justifications, Cartwright described ‘drapetomania’ as a mental illness that caused enslaved Black people to flee from their white enslavers (Cartwright, 1851: 707–8). Such racism did not remain in the South, despite the North's Civil War victory having officially ended slavery. In 1887, Northern psychiatrist Judson B. Andrews argued that emancipation had actually precipitated the deterioration of b/Black mental health, as large numbers migrated to the Northern states:

In the negro race, the proportionate increase of insanity is far greater than in any other division of the population. From 1870 to 1880 there was an increase in the census of the colored race of 34.85 per cent, while for the same period there was an increase of 285 per cent of the insane. This large multiplication has occurred since emancipation from slavery and the consequent changes in conditions and life. (Andrews, 1887: 194)

This thinking did not end with the 19th century—as Summers established in his history of race and mental illness in Washington DC (Summers, 2019)—nor even with the borders of the US. Historian Lynette Jackson has demonstrated that the sort of domestic migration that made freed African Americans so visible in the US, also rendered African economic migrants within Africa more visible and ‘more likely … to become objects of colonial psychiatric’ attention (Jackson, 2005: 69). Jackson described an out of place-ness, which drew local authorities’ attention to certain people in African urban streets (ibid.: 17, 30). Her study of the Zimbabwean (formerly Southern Rhodesia) Ingutsheni psychiatric hospital, 1908–68, noted that high numbers of male patients migrated from other African nations to find work, often in colonial seats of power. Migrant demographics—between 98.8 and 99.6% were male—were therefore mirrored in asylum admissions (ibid.: 68). Yet ‘colonial psychiatrists, amateur psychologists, and other so-called experts’ argued that these statistics, ‘resulted from their [male Africans’] greater exposure to the stresses and strains of European civilization, combined with their inability … to adjust to “culture contact” and “transition”’ (ibid.: 74). Nevertheless, Jackson's work has shown that African women were ‘difference to the second power’, or ‘the Other's Other’: doubly disadvantaged by being Black and female (ibid.: 104).

The wholesale switch in believing people racialised as black were impervious to mental illness to being especially susceptible could be swift. Take depression in Sierra Leone, West Africa, for example. Before the late 1950s,

accounts of medical practitioners … in South Africa, Kenya, and the Gold Coast, were in general agreement: depression was rare among Africans; when occurring, it was mild and short-lived; and suicide and self-denigration, elements in the depressions of European psychotics, were unusual. (Bell, 1991: 132)

Conversely, during the collapse of empires in Africa in the 1950s and 1960s, the recorded rates of depression rose significantly. In ‘one of the first English-language historical analyses of African psychiatry’ in 1991, Leland V. Bell attributed this to ‘a greater sensitivity to the condition, an increased incidence of depression, and larger numbers of indigenous personnel capable of detecting the disorder’ (ibid.: 133). However, the unseen inheritance of systemic ideological racism was at the heart of decisions around how ‘race’ was responsible for mental health.

## Questioning established aetiologies (late 19th and early 20th centuries)

In colonial asylums, staff struggled to understand aetiologies among colonised people. Around 1900, at facilities in the Natal region of South Africa, for example, ‘the cause of mental illness was said to be unknown for 37 per cent of whites … 74 per cent of “Natives”, and 78 per cent of Indians’ (who had been transported as indentured plantation labourers). This was complicated by gender, when ‘unknown’ causes made up ‘77 per cent for African women, and a whopping 90 per cent for Indian women’ (Parle, 2007: 118–19). Historian Julie Parle argued that, with the exception of attributing cannabis smoking as a cause of illness among ‘non-white’ patients—echoed in the historical aetiology of hashish and Muslims in North Africa (Keller, 2001: 49–57) and hemp in India (Mills, 2000)—the ‘origins of their madness were largely unknown to western psychiatry’ (Parle, 2007: 118–19).

Concurrently, in America, work by people of African descent pushed back against racist ideologies. Influential American Ghanaian intellectual, the sociologist and civil rights activist, W. E. B. Du Bois explored the role of racism in health and psychology. His 1903 concept of ‘double consciousness’ explained how racism shaped Black selfhood. ‘One ever feels his two-ness,—an American, a Negro; two souls, two thoughts, two unreconciled strivings; two warring ideals in one dark body’ (Du Bois, 1903: 38). Du Bois’ work centred on the economic and social context in which racism thrived. In his historical study *Black Reconstruction*, Du Bois turned his lens onto reconstruction after the Civil War. The national Freedman's Bureau initiative—to democratise wealth and to uplift impoverished people, whether racialised as black or white—was sunk by racist logic, which framed white people as disadvantaged by formerly enslaved people being given land to help secure self-sufficiency (Du Bois, 1935). Industrial capitalism necessitated keeping African Americans ‘in their place’. Europe and America's socio-economic system therefore broadcast a racially stratified hierarchy of deprivation—the 19th-century ‘less eligibility’ principle writ large globally.

Other Black thinkers soon followed Du Bois, including people entering the psy-disciplines, who took their insights and experiences into spaces of professionalised whiteness. Born in Liberia and graduating in 1904, Solomon Carter Fuller is recognised as the first Black psychiatrist in the US (Kaplan, 2005; Kaplan and Henderson, 2000; Mohammed, 2021). Steadily, the number of Black people involved in psychology and psychiatry grew, seeding challenges to universal aetiologies based on racial stereotyping. For instance, West Indian-born psychiatrist and neurologist Ernest Y. Williams graduated in 1934. His work, in part with his Black colleague Dr Claude P. Carmichael, refuted established aetiologies of African American mental illness, particularly the belief in emancipation as a cause (Williams, 1937; Williams and Carmichael, 1949). Psychiatric hospital data showed that ‘the incidence of mental disease among Negroes varies a great deal depending upon the section of the country and the proper evaluation and interpretation given’ (Williams and Carmichael, 1949: 277–8). The admission of people racialised as black with ‘certain forms of mental diseases that [were] allegedly seen more often among Negroes’ was likewise refuted (ibid.: 277). Quoting hospital statistics alongside passages by figures such as J. C. Carothers—a white, South African-born British doctor working in Kenya—Williams and Carmichael concluded, ‘Many of our scientists have not been as objective as they might have been so that even now we are not sure that the data presented were objectively reported’ (ibid.: 281).

Consolidating work around ‘the African mind’ arose between the renewal of imperial power, around the time of the First World War, and when later empire was under sustained threat. Carothers' view, alluded to above, was part of the foundational thinking at the newly formed World Health Organisation (WHO), which invited him to write a report in 1953. He wrote,

The African … has been described as conventional, highly dependent on physical and emotional stimulation; lacking in spontaneity, foresight, tenacity, judgment, and humility, inapt for sound abstraction and for logic, given to phantasy and fabrication, and in general, unstable, impulsive, unreliable, irresponsible, and living in the present without reflection or ambition, or regard for the rights of people outside his own circle. (Carothers, 1953: 87)

Carothers also penned *The Psychology of the Mau Mau*, about the Kenyan resistance fighters to British rule. Commissioned by the British government, he was tasked with explaining the psychology behind the rebellion. Drawing extensively on his WHO report, Carothers offered a medicalised solution for what was seen as Mau Mau violence (Carothers, 1954).

In the 1950s, two Black psychiatrists challenged racist ideas about mental health and developed decolonial praxis: Frantz Fanon (1925–61) and Thomas Adeoye Lambo (1923–2004). Fanon, a psychiatrist and philosopher, has become a pivotal figure in both transcultural psychiatry and postcolonial theory. The book that cemented his reputation, *Black Skin, White Masks* (1952), reflected his own experiences of discrimination as a Martiniquan emigrating to the French metropole to complete his psychiatric training (Robcis, 2021). Drawing on psychoanalytic theory, Fanon argued that colonialism was not only political but also psychological subjugation, which coerced colonised individuals to enact ‘whiteness’, using the language of the coloniser to integrate. This required the suppression of Black identity and tradition, and the repression of the trauma that these colonising processes caused, resulting in mental illness. White domination of the psy-professions, he argued, also meant that clinical theories and practices failed to recognise the experiences of patients racialised as ‘non-white’ (Fanon, 1986[1952]). At the psychiatric hospital in Blida, French Colonial Algeria, Fanon developed culturally sensitive approaches to Arab patients, who, he observed, were not engaging well with clinical practices rooted in French assumptions (Fanon and Azoulay, 2018[1954]).

A contemporary of Fanon, the University of Birmingham-trained Nigerian psychiatrist Thomas Adeoye Lambo, also developed localised approaches. He established the Aro Village System of community psychiatry, where patients stayed in villages close to the Nigerian hospital he directed. Emphasising this setting as more culturally appropriate to Africans than inpatient care, Lambo introduced a system by which village elders managed psychotherapeutic and electroconvulsive treatments alongside traditional interventions by ‘native healers, diviners, [and] witches’ (Osborne, 1969). Naturally, in so large a continent, ideas in Africa about causation were diverse. Around the Aro villages, there was a local conceptualisation of *wére*, the Yoruba term often translated as ‘madness’, though not mapping neatly onto ‘Western’ ideas (Nabel, 2017: 6). Here, as in other African nations, Parle noted that traditional social anxieties were articulated in ideas around possession and witches. Moreover, she emphasised, these too were in flux during the decades around the turn of the 20th century, and the historiography of African aetiology is limited (Parle, 2003). Like Fanon, Lambo argued that through racist ignorance, colonial psychiatry did not understand African subjects (Heaton, 2018). Cases of psychosis, especially among the Yoruba, had gone unnoticed by British doctors, who failed to distinguish between everyday local beliefs in witchcraft and delusions based on them (Lambo, 1955). He also argued that colonial psychiatry incorrectly, and wilfully, ignored the prevalence of depression in African patients as a result of racist assumptions about their primitive and ‘care-free’ nature. In so doing, the colonisers justified the neglect of mental health care in Nigeria, using psychiatry as a tool for criminalisation and control of the local population, and worsening Africans’ mental ill health (Heaton, 2018).

The same sort of racist consolidation seen in colonised Africa happened in the US during the 1950s and 1960s civil rights era, as psychiatrist-historian Jonathan Metzl has so effectively demonstrated—but, in this case, diagnostic categories were used excessively to stigmatise and control Black populations (Metzl, 2010). Context here altered diagnostic demographics: by the mid 1970s, schizophrenia shifted from ‘an illness that afflicted nonviolent, white petty criminals [and …] women from rural Michigan’ to become ‘disproportionately applied to … [mainly] African American men from urban Detroit’ (ibid.: xv–xvi). As suggested in relation to depression, aetiology is directed by stereotypes. Aided by the American Psychiatric Association's 1968 *Diagnostic and Statistical Manual* (*DSM*), behaviours that white America read in the Black Power movement—belligerence, Muslim practice, interest in Africa—came to be ascribed by psychiatrists as ‘protest psychosis’ (ibid.: 98; ch. 13). Metzl argued that though descriptions of symptoms deliberately moved schizophrenia from women and attributes perceived as female, the *DSM* reflected its racist context, which ‘enabled users to knowingly or unknowingly pathologize protest as mental illness’ (ibid.: 98). ‘Careful investigation of the mental content’, wrote Walter Bromberg and Franck Simon, the two psychiatrists who coined ‘protest psychosis’,

demonstrates antiwhite productions and attitudes.… It becomes apparent that the intellectual dissociation represents in part a refusal to accept the syntactical language of standard English.… Often the prisoners draw pictures or write material of an Islamic nature, elaborating their ideas in the direction of African ideology with a decided ‘primitive’ accent.… Islamic names are adopted or they change them in such a way so as to deny the previous anglicization of their names.… Bizarre religious ideas are Moslem in character. (ibid.: 101)

For both Carothers and those behind the inscription of schizophrenia with Blackness, the notion of ‘culture’ was itself subsumed into discriminative praxis, as we shall see. But ‘the monologue of European reason about African [and Black] madness’ (Jackson, 2005: 105) was, nevertheless, interrupted by new voices.

## Universality and unpicking prejudice, 1945–90s

Social psychiatry gained global traction in the 1940s, assisted by ideas about prevention and the success of psychotherapy for frontline Second World War service personnel. The conflict also demonstrated that a wide variety of people could enter a battlefield without mental health difficulties and leave with them, universalising experiences and responses, and suggesting that such issues were considerably more widespread in the population than institutional statistics suggested. William Menninger, a wartime psychiatric adviser to the US Army, said that ‘Millions of people became really aware for the first time, of the effect of environmental stresses on the personality’ (Menninger, 1948: vii). What this meant for advocates was both the eschewing of narrow biological causation and the rejection of artificial and racist distinctions between brains and minds. Black people were thus open to new avenues of aetiology and therapy that had previously been the preserve of people racialised as white: all forms of ‘mental disorder’ could now, theoretically at least, be ascribed to all ‘races’.

With others, German Jewish psychiatrist Frederic Werther established an anti-racist psychotherapeutic clinic in 1946 in Harlem, a traditionally African American district of New York. Werther argued that staff must ‘understand a patient's personal and biological history to get at the micro-dynamic factors in his case, and his economic position and group culture to grasp the macro-dynamic factors’ (Doyle, 2009: 755). In the 1954 inaugural issue of the *International Journal of Social Psychiatry*, Thomas Rennie, the first professor of social psychiatry in the US, was even clearer about ascertaining the causes of mental ill health (Smith, 2023: 18). Social psychiatry, he argued, ‘is concerned not only with facts of prevalence and incidence, it searches more deeply into the possible significance of social and cultural factors in the etiology and dynamics of mental disorder’ (Rennie, 1954: 10). The discipline ‘is etiological in its aim, but its point of attack is the whole social framework of contemporary living’ (ibid.: 12), including a vast ‘cultural matrix’ embracing cultural inheritance and relations between groups (ibid.: *passim*; quote from 11). That traces of psychodynamic conceptualisation and social psychiatry should be embedded within the US *War Department Technical Bulletin, Medical 203* was no coincidence. Issued by Menninger's office, *203* was ‘the first dynamic diagnostic nomenclature for psychiatric disorders. It reflected psycho-analytic concepts of personality development and presented mental illness as stress reactions manifested by symptoms. This nomenclature was later to be adopted by the American Psychiatric Association’ as the first *DSM* in 1952 (Barton, 1987, quoted in Houts, 2000: 944).

This switch in emphasis away from race and towards culture and social relations is discernible through the *DSM*'s 20th- and 21st-century incarnations. Despite the foreswearing of causation, aetiological ideas were evident through its language and framing. Whereas *DSM-I* (1952) did not mention culture, *DSM-II* (1968) began to recognise its role in aetiology: those who were considered to be exhibiting ‘social maladjustment’ had been ‘individuals thrown into an unfamiliar culture (culture shock) or into a conflict arising from divided loyalties to two cultures’ (*DSM-II*, 1968: 51–2). The 1980 *DSM-III* expanded slightly its discussions around culture, especially regarding the content of delusions and judgements of sexuality. From this edition's inclusion of 14 allusions to ‘culture’ (*DSM-III*, 1980), however, *DSM-IV* (1994) mentioned culture 216 times, and *DSM-5-TR* (2022), 267. This rise in cultural awareness, while valuable for developing a more sensitive psychiatric profession, also meant that prejudice was baked into assessments of cultural difference, as presaged by earlier judgements around spiritual beliefs and drug practices.

From the 1940s, ‘transcultural psychiatry’ centred on anthropologically informed explorations of culture. Just as the *DSM* aimed to standardise psychological diagnoses, so efforts by new global organisations (WHO and UNESCO) were influenced by transcultural psychiatry and sought to equalise peoples across the world. By this alone, it is possible to see how the doublethink around ‘race’ was embedded in new systems. This ‘shift away from Eurocentrism towards a world of separate but interrelated civilisations’ (Betts, 2020: 319–20), Antić has argued, ‘coexisted with, or even encouraged, Western interventionist modernization projects in the Global South and fostered perceptions of non-Western communities as ‘backward’, ‘primitive’ and in need of rescuing’ (Antić, 2022: 27).

Established expressly as an interdisciplinary approach, the first dedicated transcultural psychiatry department was founded at Canada's McGill University in 1955 by Eric Wittkower and Jacob Friend, linking the faculties of psychiatry, sociology, and anthropology. Joining them in 1959, psychiatrist Henri Ellenberger defined ethno-psychiatry as ‘the study of mental illness according to the ethnic or cultural groups to which patients belong’ (Ellenberger, 2020[2017]: 131). As a discipline, it recognised that textbook descriptions of mental illness were based solely on the examination of ‘Western’ patients. By studying more diverse groups, Ellenberger contended, descriptive diagnoses would be enriched, unusual forms of disorder specific to certain cultures comprehended, and ‘Western’ doctors would better understand and treat patients from other backgrounds. Most importantly for this article, he and his colleagues believed that exploring the comparative frequency of diagnoses in different nations or groups would help researchers study the role of ethnic and cultural factors in the causation of mental illness.

Indeed, *DSM-IV* incorporated the ‘culture-bound syndrome’ element of transcultural psychiatry (Delille, 2020[2017]: 76). However, while transcultural psychiatrists believed that such syndromes could arise in *any* culture, the *DSM* focused on ‘non-Western’ examples. This implied that culture was an influence on mental ill health only when it was other-than-Western, and also that the *DSM* (in which descriptions remained based on ‘Western’ patients) was generally universal and *not* culture-bound.

In fact, the path taken by transcultural psychiatrists was itself directed by local culture, so whereas the US concentrated on the homogenisation of disease concepts across racial categories (predominantly determined by privileged white men), in the UK attention turned to race and racism as a reflection of post-war immigration patterns (Bains, 2005). HMS *Windrush*'s 1948 arrival in Britain marked the start of this change, bringing workers the UK needed at a point when anti-colonial thinking flourished and the British Empire was collapsing. Against this complex backdrop, and the radical shifts of the 1960s, a new generation of psychiatrists racialised as ‘non-white’, trained in the UK and experienced its overt and structural racism. At the vanguard of such UK-based psychiatrists were Aggrey Burke and Suman Fernando. The group in which they were key actors, the Transcultural Psychiatry Society (TCPS), was collectively important. Developing from Morris Carstairs and Philip Rack's 1976 International Congress on Transcultural Psychiatry, TCPS was founded by John Cox and Sashi Sashidharan (the latter long associated with Birmingham). The group (laid down in 2008) was the UK ground zero for the theory that racism itself caused mental health difficulties. For many of those affiliated with TCPS, historical knowledge was essential—through it the underlying, unseen and often unchallenged ideas of ‘civilisation’ and ‘barbarity’ were revealed. In the seminal book, *Aliens and Alienists: Ethnic Minorities and Psychiatry*, the anthropologist-psychiatrist team of Roland Littlewood and Maurice Lipsedge extensively predicated their discussions on the history of medicine and racism (Littlewood and Lipsedge, 1989). This linkage, the need to understand history in order to understand mental ill health, was central to the lifelong work of (trans)cultural psychiatrists Frederick Hickling, Aggrey Burke, and Suman Fernando.

### Frederick Hickling

Hickling was born in 1944 to professional parents in colonial Jamaica. Anne Hickling-Hudson, a trained historian, pioneering educationalist, and Frederick's sister, recalled that they ‘absorbed the oral history of our foreparents’ at a point when Jamaica was ‘steeped in conservative colonial thinking’ (Hickling-Hudson, 2020: 148–9). The great-nephew of the first Black psychiatrist in Jamaica (Hickling, 2007: 3), Hickling enrolled at the University of the West Indies (UWI) on the eve of the island's 1962 independence from Britain. Independence had loomed large in Hickling's life for some time, in part because he was a ‘gofer’ to members of the official celebration committee, which led to his sustained involvement with the National Dance Theatre Company (ibid.: 4–5). Overlapping with this time, he received ‘specialist training in anatomy at St. Thomas’ Hospital Medical School, University of London, and postgraduate training in psychiatry at UWI and the University of Edinburgh’ (University of the West Indies, 2017). Hickling was appalled by open racism in the UK (Hickling, 2016). Returning home, and inspired by Fanon, Hickling encountered both historiography, through Guyanese historian Elsa Goveia—and the community psychiatric movement (Hickling, 2007: 9).

The stage was set for Hickling's innovations at Kingston's Bellevue Hospital in Jamaica, once he was appointed there in 1973. His reading of English asylum psychiatrist John Conolly's 1847 description of Bellevue (Conolly, 1847: 82–3)—which Hickling recalled as stating that it was the ‘prototype for mental hospitals across the British Empire’ (Hickling, 2007: 10)—and his encounter with the reality of the colonial psychiatric hospital, consolidated his lifelong lodestar. In ‘postcolonial Jamaica’, he later reflected,

our greatest mental health challenge has been to counter the psychological impact of 500 years of European racism and colonial oppression. For the descendants of African people enslaved in Jamaica, this has been a process of dismantling the colonial policies and structures that saw thousands of Jamaicans incarcerated in lunatic asylums, while also seeking ways to heal the historical legacy of sustained structural violence and abuse that remain potent causative factors in contemporary mental illness. (Hickling, n.d.)

From the 1970s, Hickling's aim for Caribbean deinstitutionalisation depended on the mental health of those inside and outside Bellevue. His experiences, beliefs, and inspirations coalesced in the development of ‘psychohistoriographic cultural therapy’; as history was the cause of mental illness, so confronting this history and working through it would decolonise patients and communities psychologically. Psychohistoriography ‘combines historiography … with the oral tradition of verbal anecdotes’ (Hickling *et al.*, 2010: 137). In essence, this group therapy sought ‘psychic centrality’ by ‘creating a historical map of collective experience’ while also working through personal histories. The map informed creative therapy and helped develop performances or ‘pageants’, which were held at a specially built public theatre. In this way, colonial history was seen as a cause of group dynamics and mental health challenges and able to be addressed (ibid.: 136). Begun in 1978, this ‘sociodrama, a synthesis of group psychotherapy and theatrical presentation’ (Hickling, 1989: 402), was introduced at Bellevue and ran until 1981, with the therapy going on to be applied in Grenada, the US, Belize and Canada (Hickling, 2004; Hickling *et al.*, 2010).

Within this overarching framework of historical-racism-as-cause, Hickling, with Gerard Hutchinson, hypothesised about the weight of racism, history, and colonisation on the African Caribbean psyche. In the same way as, 100 years apart, Du Bois and Harewood observed the internal tension of this two-ness, Hickling and Hutchinson argued it created ‘roast breadfruit psychosis’. This term ‘[referred] to people who though Black skinned, see and identify themselves from a White and Euro centric perspective’ (Hickling and Hutchinson, 1999: 132). The 1948 opening of the UK to Caribbean migrants, they argued, meant that the ‘rationalisation of identity may be central to the reports of high rates of psychosis in Britain, especially when combined with socio-economic deprivation’ (ibid.: 133). In contrast to this ‘epidemic of schizophrenia’ among African Caribbean people in the UK, Hickling's 1996 ‘Jamaican study [represented] the only study of White emigrants to a Black country, and, according to Hickling and Hutchinson, demonstrates that wherever White people are in the former colonial world, they carry the power and sense of ownership of their environment that has been incorporated into their being. This may act as a protective factor from psychopathology’ (ibid.: 132–3; Hickling, 1996).

### Aggrey Burke

Burke was born in Jamaica in 1943 ‘to politically active parents’ (Vernon and Osbourne, 2020). His father, Reverend Edmund Burke, was sent to the UK to improve ‘race relations’ after anti-b/Black violence erupted in 1958. Aggrey Burke grew up surrounded by racist and racialised ‘judgements made about African Caribbean people and the practical disadvantages they suffered, which contributed to many forms of discomfort of *being*’ (ibid.; original emphasis). Around a decade after Lambo had left the city and 30 years before Hickling spent time there, Burke enrolled in the University of Birmingham's medical school in 1962. He travelled to Trinidad and Tobago for his psychiatric training. Returning to Birmingham to complete this, he moved from a research fellowship to St George's Hospital, London, becoming the first Black forensic psychiatry consultant in the NHS.

Building on 1960s studies, Burke explored ‘the relative importance of environmental and genetic factors in mental illness’ by researching migrants (Burke, 1973: 109). Working with the large West Indian, Asian, Irish, and Commonwealth migrant communities in Birmingham between 1969 and 1972, Burke found them more likely to attempt suicide than in their home countries (Burke, 1976a, 1976b, 1976c, 1978). In considering the limited literature on Black suicide, Burke's selection reflected the impact of racism within US aetiological discussions. These varied from ‘Aubin [noting] that suicidal behaviour was commonly carried out by African slaves who were attempting to escape from torture (Masaryk, 1888)’; to ‘Prudhomme (1938) [believing] that the black race was neurotic and therefore had an attempted suicide rate which was as great or even greater than that of the white race’ (Burke, 1976c: 261). Confounding this story, Burke also consistently found that rates were lower in immigrant populations than among white ‘natives’ (ibid.: 264).

Following his move from Birmingham to London, Burke's focus (and that of the TCPS) altered, asking ‘Is racism a causatory factor in mental illness?’, with the answer a firm and consistent yes. The shift occurred as a result of events in 1981 (Burke, 1984a), including the New Cross Massacre and the uprisings in Brixton, Handsworth, and elsewhere. The fire, suspected to be racist arson, at a 16th birthday party killed 14 Black people and galvanised the Black People's Day of Action, ‘a pinnacle in the organising power of black radical activists in Britain’ (Waters, 2019: 215). These events are widely considered to have helped theorise, consolidate and mainstream Black British identity (Andrews, 2021; Waters, 2019). Just as Black psychiatrists had done previously, lay activists ‘[linked] black history to black future’ (Andrews, 2021: 184).

The events catalysed TCPS activities. As a member of TCPS and the New Cross Massacre Action Committee, Burke's psychiatric skills brought him into close contact with New Cross survivors, their families, and the local community—indeed, he was part of a ‘rescue group for the bereaved and families of the injured’, of Black social and community workers, psychologists, and nurses (Burke, 1984b: 65). In the aftermath of 1981, Burke established ‘The Ethnic Study Group’. According to member Hari Maharajh, the multicultural discussion group ‘determined that diagnoses such as ‘Balham psychosis’, ‘New Cross psychosis’, ‘West Indian psychosis’ and ‘Migration psychosis’ were nonsensical in nature. These were interpreted as intentions to create nosological entities that did not exist, while simultaneously attempting to negatively categorise Black people in Britain by imposing prejudicial values based on psychiatric imperialism’ (Maharajh, 2000). The Ethnic Study Group and TCPS connections led to a joint meeting in 1982, which in turn resulted in a special issue of the *International Journal of Social Psychiatry* edited by Aggrey Burke in 1984. Here Burke argued that ‘as a result of racism it will be found that there will be parallel psychological mechanisms and psychological problems in the subjected group … the appropriate terminology’ for such mental health issues ‘is racism-related disorders’ (Burke, 1984a: 1). He also ‘noted that psychological disturbances due to racism were distinct from those caused by cultural factors’ (Maharajh, 2000: 97). He understood the disadvantage West Indian people faced was not simple, but comprised an interplay of variables. ‘The variable of race is relevant in so far as it increases the risk of exclusion of this group already tainted by ‘madness and badness’.… This issue of exclusion may be a function of cultural factors that lead to social stigma as well as racial factors affecting the total community’ (Burke, 1986: 185).

In 1984, Burke reflected on his work with Black communities in Birmingham and London: the ‘medical model of madness … suggests that the mentally ill have a basic organic impairment mediated by genetic factors and with some structural brain abnormality yet to be identified. Is it not true that blacks have been similarly described?’ (Burke, 1984b: 50). His focus on grief and loss is unsurprising. As discussed earlier, racist stereotypes often framed Black people as being impervious to sadness, which had meant many psychiatrists viewed people of African descent as unlikely to experience depression. However, it is also possible to see how Burke's reading of history was reflected in his ideas about how psychology was shaped by culture. He drew on James Walvin's seminal *Black and White: The Negro and English Society, 1555–1945* (one of the first substantial, scholarly histories of Black people in Britain). Walvin argued that between 1900 and 1914, people racialised as black ‘were, like so many of their unknown ancestors, depressed people, eking out a living on the poverty-stricken fringes of society’ (Burke, 1984b: 50; Walvin, 1973: 202). Burke the psychiatrist seems to have read this as a state of mind, so that not only had depression as a psychological condition become an enforced part of Black Britons' culture via the imposition of penury, but that ‘the devastation of the many’ was caused by ‘racism … as an integral part of European culture’ (Burke, 1984b: 50). The interplay of cultures shaped by whiteness was a core theme of Walvin's historical book and of Burke's psychiatric work. By considering the experiences of depression in Birmingham and grief in London, and taking in the history of Black presence, Burke asserted that ‘depression and grief may result from an ongoing stressful life situation’ caused by racist experiences. By outlining these examples, he hoped that ‘the reader [would] begin to understand how social and psychodynamic factors interact with biological components of mood’ (ibid.: 55).

### Suman Fernando

Fernando was born in 1932 in Sri Lanka, then known by its British colonial name, Ceylon. His grandfather and father were politically active medical men—the former was one of the first South Asians to train at the University of Edinburgh in the 1870s; the latter, having studied at Cambridge in the 1900s, returned home and founded the Ceylon Labour Party. Fernando followed in their footsteps, enrolling at Cambridge in 1950 and taking up clinical training in London. After working in psychiatry in Sri Lanka, he returned to England in 1960, where he married Frances, a Jewish woman. This, coupled with Fernando having worked at London's Jewish Hospital, channelled his earliest research, on depression and Jewish people (Moodley and Ocampo, 2014). His main findings were that marginalisation played a key role. ‘Repressed anger’ manifested ‘as extra-punitive hostility among Jewish depressives’. Their ‘relatively high aspiration levels … were culturally determined and likely to cause depression if unfulfilled’. Fernando ‘noted the need for caution in applying a classificatory system cross-culturally. Thus, although British Jews and Protestants have a similar cultural and religious heritage, they were different when depressed’ (Fernando, 1988: 71).

Fernando stated, ‘What really brought me to look closely at race and culture in psychiatry was when I discovered that psychiatry in [the] UK, [has] often and quite consistently been felt by people identified as “black people”, as oppressive and discriminatory’ (Williams, 2012: 168). Given his South Asian background, the observation seems to have been especially instructive for Fernando. His involvement with the TCPS at the ‘high point’ of its activities in the 1980s and early 1990s was pivotal to his life's work (ibid.), which came particularly to centre on the racism of psychiatry. When Burke was appointed TCPS chair in 1984, Fernando became secretary. The same year, his article ‘Racism as a Cause of Depression’ was part of the *International Journal of Social Psychiatry* special issue. ‘In dealing with depression among people who are victims of social condition be it racism or unemployment’, wrote Fernando, ‘it is all too easy to see the individual as the problem. We then see solutions merely in terms of changing or treating the individual and really get into quite a mess’ (Fernando, 1984: 47). Indeed, in his first book, *Race and Culture in Psychiatry*, Fernando argued that ‘a psychiatric diagnosis should be seen as part of a statement about a patient in a social context rather than a designation for an attribute of peculiarity within the patient’ (Fernando, 1988: 183).

The 1988 text was ‘about the discipline of psychiatry [in Britain]—about the assumptions and ideologies that determine the ways in which psychiatry goes about its business in both developing theories and dealing with people’ (Fernando, 1988: xi). Resting on the early historiography of the history of psychiatry (including Andrew Scull and Vieda Skultans), Fernando firmly rooted his book in history, and systematically interlaced the emergence of the discipline from the 16th century to the 20th with the history of racism, thoroughly correlating key dates from both to argue that psychiatry was a product of ‘political and social forces’ (ibid.: Introduction and ch. 1, esp. 35–6; quote from xvii). Deconstructing these histories, he suggested, was the first step towards identifying integrated racist thinking within the culture of psychiatry, from which he could then set out a blueprint for an anti-racist psychiatry. Fernando has always and consistently argued that knowing the past is essential to understanding the causes of mental illness and therefore vital to mental health research, science, and praxis (see, for instance: Fernando, 1988, 2010[1991], 2011, 2017). Indeed, *Institutional Racism in Psychiatry and Clinical Psychology* (2017) was published in Palgrave Macmillan's Contemporary Black History series. His work has retained the echoes of his 1988 book and its blueprint, which offered practical changes to all incarnations of psychiatry, from the Royal College of Psychiatrists to medical judgements around dangerousness. In between these aspects, he challenged the systemic racism present in professional psychiatry, including academic publishing and ‘grant-giving organisations’. ‘Colour-blind[ness]’ in funding bodies should be challenged by integrating ‘a policy on race and culture’ that included ‘guidance on … the qualities necessary in researchers who are deemed suitable to carry out the research.… They should ensure that all research projects that involve black and ethnic minorities take on the racial dimension in terms of its effect on researchers’ attitudes and misconceptions determined by the racist context of society, and, the fact that racism is a major cause of social stress to black people’. While there was no advocation for embedding researchers racialised as ‘non-white’, the blueprint directly called for the eradication of ‘racism in the admission to training courses and to professional appointments in the psychiatric services’ (Fernando, 1988: 174–6).

Such structural racism was firmly evidenced by Burke and Joe Collier in 1986, when they blew the whistle on selection practices at their employer, St George's Hospital, London. They contended that the medical school selection process was racist and sexist, with those from minoritised groups significantly less likely, statistically, to be admitted. They argued that

admission arrangements at some of the London medical schools have failed to provide the equal opportunities defined by the Race Relations Act (1976). This act makes it unlawful … for an educational establishment to discriminate on racial grounds as regards term of admission for the establishment by refusing, or deliberately omitting to accept, an application for admission. (Collier and Burke, 1986: 89)

With the cultural background of practitioners so skewed, it was unsurprising that a 1990 study assessing British psychiatrists for racism concluded that ‘race-thinking is a wider problem, and though less obvious and obnoxious than outright ideological racism, it is still an unseen and damaging influence’ (Lewis, Croft-Jeffreys, and David, 1990: 415). The findings made clear that the aetiology and diagnosis of Black men (as having schizophrenia or cannabis psychosis) were more likely to be influenced by racial stereotyping (ibid.). This same ‘race-thinking’ was evident among psychiatrists elsewhere. For Hickling, the concerns raised by ‘worldwide psychiatric challenges are often viewed through first world spectacles, which ignore the perceptions of the rest of the world, and [are] often sidelined as transcultural psychiatry’ (Hickling, 2009: 67).

## Individualising interrupted, c.1990–2020

Meanwhile, in the 1990s biomedical explanations for mental illness dominated. A new hypothesis emerged from animal models. Scientists designed experiments to cause conflict by inserting one rodent in the cage of another. They observed that the animals, especially individuals forced into a submissive situation, manifested signs of chronic stress. This ‘social defeat’ seemed scientifically to develop the sorts of assertions that had been made by the humanities, social sciences and transcultural psychiatry: persistent social stress impinged on individual brain function. Observed responses in mice have suggested an association between social stress and depressive or anxiety behaviours (Lehmann *et al.*, 2020). In some ways universalism was challenged by new models such as social defeat, which centred individual responses to outside stressors such as bullying, hopelessness, aggression, and violence (Allen and Badcock, 2003; Bjorkqvist, 2001; Rohde, 2001)—and therefore racism. This model could be read as blaming the individual for the way they reacted, not the stressors themselves.

This echo of late-stage capitalism, by which structural issues are ignored and individual fault centred, could, however, be integrated in mental health research to different effect. For anthropologist Tanya Luhrmann, the concept of social defeat saw science catching up with the humanities and social sciences in seeing the social causes of mental illness, including a historically shaped environment that manifested significant challenges and bred ‘[constant] small defeats’ (Luhrmann, 2007: 157). ‘And so to look into the eyes of a homeless psychotic woman in Uptown [Chicago], is to see not a broken brain, but a social history’ (ibid.: 163). Indeed, part of the bedrock of Luhrmann's assertions was the 1990s and early 2000s reframing of migrant and ‘non-Western’ health issues to balance individualism in immigrant and host-nation inhabitants. For example, while recognising reception could vary by location, Dinesh Bhugra and Peter Jones noted that

migration remains an enigma for the clinician because not all migrants go through the same experiences and/or settle in similar social contexts. They do not all prepare in the same way and their reasons for migration are variable. The process of migration and subsequent cultural and social adjustment also play a key role in the mental health of the individual. (Bhugra and Jones, 2001: 221)

Beyond discussions of the pathway of migration, the aetiological promise of ‘the coming together of developmental neuropsychology, cognitive science and social anthropology, and the contemporary interest in consciousness’ (Dein, 1994: 562), was realised in part by the Aetiology and Ethnicity in Schizophrenia and Other Psychoses (AESOP) study. Established in 1997, AESOP aimed ‘to investigate the high rates of psychosis in African-Caribbean populations from the UK, and from this to shed light on the aetiology of psychosis in general’ (Morgan *et al.*, 2006: 40). Emphatically drawing on comparative studies since the 1960s, research was carried out in London, Nottingham and Bristol and was designed to avoid previously criticised methodological issues, including the use of inaccurate statistics, in part by employing a range of clinical, psychosocial and cognitive/biological data collection techniques (ibid.: 40, 43). Geography, their findings suggested, altered aetiology (ibid.: 42), yet ‘the 3-fold increased incidence of psychoses in the BME [‘Black and minority ethnic'] group compared with the white British group [… was found] across study centers and broad diagnoses’ (Kirkbride *et al.*, 2006: 255). The researchers argued that neither diagnostic bias nor localised racial and ethnic demographics explained the differentials (ibid.: 250, 255). Environment and ‘population stratification’ suggested ‘truly “psychotogenic” effects … in terms of psychosis risk’ (ibid.: 255). In short, despite the vaunted bells and whistles of modern mental health science, the findings concluded that acculturation and social ‘[disadvantages] may combine in complex ways to push some individuals along a predominantly socio-developmental pathway to psychosis’ (Morgan *et al.*, 2014: 407). Along with other studies—not least those that found rates of diagnosis and recovery in India and contrasted extremely well with nations framed as ‘developed’ (e.g. Craig *et al.*, 1997; Hopper *et al.*, 2007; Thara and Eaton, 1996)—AESOP contributed to the recognition that there was not some grand global psyche to which everyone was equally subject (Morgan *et al.*, 2006: 43).

The danger then as now for individual Black patients, especially migrants to what are termed ‘Western countries’, is that aetiologies differ across borders. For psychiatrist and medical anthropologist Arthur Kleinman, ‘explanatory models’ described the constellation of factors that align to define health, manifest sickness, and decide treatment (Kleinman, 1980). As psychiatrist Vikram Patel argued in relation to Sub-Saharan Africa, illnesses conceived as ‘of the “spirit” and of the “soul” were probably analogous to mental illness’, with the somatic seats of mind scattered between the head, chest and abdomen (Patel, 1998: 15). For him, the 1987 findings of transcultural psychiatrist Frank Kortmann—that mental illness in Ethiopia excluded many less severe forms of psychiatric disturbance that were not considered as being illnesses (Kortmann, 1987)—has ‘echoes of studies from other African cultures’, as well as Caribbean (Fisher, 1985: 58–63; Patel, 1998: 16).

Such misunderstandings (wilful or lazy) of ‘non-white’ cultures in ‘Westernised’ thinking suggest that, far from the individual fault or aetiology present in Mental Health Science, the issues are group-wide, social and systemic, as the surge of papers from the 2010s argue. Racial trauma, a form of race-based stress [… describes] reactions to dangerous events and real or perceived experiences of racial discrimination … [it] is unique in that it involves ongoing individual and collective injuries due to exposure and re-exposure to race-based stress[.… Similarly,] historical trauma or soul wounds—the cumulative psychological wounds that result from historical traumatic experiences, such as colonization, genocide, slavery, dislocation, and other related trauma—… can have intergenerational effects. (Comas-Diaz, Hall, and Neville, 2019: 1–2)

The insights and understanding opened up by attention to social and cultural history have been integrated into psychiatry by some practitioners, but not so far into mental health science. This sort of loss has rendered ‘observers’ unable ‘to view other types of racism and ethnoviolence as life-threatening (e.g. vicarious experiences, exposure to microaggressions) because the historical roots of the trauma are invisible’ (Helms, Nicolas, and Green, 2012: 65).

Understandings of racism in causation therefore require history, and this includes personal histories. As someone with lived/living experience put it when they were asked in a survey about the causes of mental ill health: ‘I didn't have mental health problems until I experienced abuse and sexual violence. It might be called PTSD, but it's really a normal reaction to racism and rape’ (Marks, Chaney, and Wynter, 2023: 10). Diverse representation among the professional and public groups operating in mental health policy, research, and science, then, is key to ensuring that knowledge and insights acquired through Black experiences, as well as those of other minoritised groups, are integrated into dialogue, decision-making, and action. UK equality, diversity, and inclusion programmes have contributed, but the push for involvement has often come from Black communities and from survivors/service users. Canerows, for instance, was established in London in 2007 by service users Devon Marston, Coral Hinds and James Braithwaite, ‘who were motivated by their own experiences to improve the experience of BAME [‘Black, Asian and minority ethnic’] people on inpatient wards’ (Faulkner, n.d.: 1). Their aim was to introduce peer support to the wards of South-West London and St George's Mental Health NHS Trust (Reynolds, 2010), where Burke had worked and written about in the 1980s. ‘Canerows represented something of a challenge to NHS culture, which was hard for the service to accept at first’ (Faulkner, n.d.: 2).

Developing from these sorts of interventions, the NHS today employs mental health peer support workers (NHS Health Careers, n.d.). Although this is an example of how lived/living experience has impacted on policy and practice, the integration of Black service users in mental health science is only now growing, with funding bodies’ integration of public and patient involvement (PPI). However, PPI-informed research has tended to focus on pathways to clinical care (Bowl, 2007; Mclean, Campbell, and Cornish, 2003; Rabiee and Smith, 2014). Co-production has increased, though this too has proved problematic for genuine collaboration (Patterson, Trite, and Weaver, 2014; Rose and Kalathil, 2019). People racialised as black are still under-represented in the public involvement communities that shape health and social care research (NIHR, 2019; Shimmin *et al.*, 2017). Black and ethnically minoritised staff are also under-represented in the public engagement workforce—those whose roles are centred around having, or facilitating, inclusive conversations about science and research (Heslop, 2019). This lack of representation in the workforce may be a contributing factor to that found in PPI. Indeed, according to 2015–20 ‘ethnic minority’ statistics from UK Research and Innovation, the central government funding body for research, for example, people racialised as black formed the smallest percentage of applicants and were among the lowest percentage of awardees (UKRI, 2021). This reflects figures from UK Higher Education, which demonstrate their under-representation in student and academic bodies (Gibney, 2022). In other words, society continues to oppress and suppress Black participation.

Initiatives, however, began to appear as part of the global response to George Floyd's 2020 murder and the global mainstreaming of Black Lives Matter. In the UK, these programmes aim to encourage diversity in clinical and academic research and include better support for Black researchers (UKRI, n.d.; Wellcome, 2023); the development and sharing of resources to support researchers to involve Black communities (Centre for Ethnic Health Research, n.d.; Learning for Involvement, 2021); and notable examples of mental health research expressly co-produced with members of the Black community (Centre for Mental Health, 2022). Yet there is still so much further to go—and, as we have all recently witnessed, support for social justice can be so fragile in the face of racism.

## Conclusion

Historical globalised structures and ideas continue to shape thinking about minds, brains, and aetiology. Terminologies shift, but remain rooted in the past and perpetuate attitudes, and are consciously deployed by racists—see civilised, industrialised, developed, Western, versus primitive, simple, developing, other cultures. Uncritical use of statistics and other scientific methods means that the risk of past patterns of racialisation is very present—this is especially true when it comes to thinking about drug use, such as cannabis, in aetiologies of mental ill health, and in ascribing ‘Western’ models and theories to different cultures. Globalising mental health science and research is laudable, but could offer a new form of colonisation and the sort of universalism that saw the dominant narrative shift so dramatically—from the idea that b/Black people were impervious to mental illness, then especially susceptible, to equally affected—and now potentially to attributing all mental ill health in certain groups to racism. This universalising has historically been at worst violently racist, and at best a red herring.

Transcultural psychiatrists have emphasised the importance of understanding historical processes and legacies of colonialism for the contemporary moment. The repeat of patterns exacerbating mental health issues for those racialised as ‘non-white’ continues to be part of today's transcultural and cultural psychiatry discussions, with Kamaldeep Bhui and Kwame McKenzie being two leading thinkers. That is not to say ‘nineteenth-century British racism in the colonies’ presented in the same way as ‘current manifestations of racism in the UK’ (Fernando, 2011: 46). But until mental health science and the clinical professions truly address equality, diversity, and inclusion within their own ranks, they will remain discriminatory spaces that fail properly to address the mental health needs of minoritised and racialised communities and therefore of entire societies.

This article has traced the history of ideas around colonialism, discrimination, racial exclusion, and anti-b/Blackness as central aetiological factors in mental ill health. It has foregrounded the work of a range of progressive transcultural psychiatrists and psychologists from the mid 20th century to the present, who worked to understand the mechanisms by which racism has caused psychological harm. Substantial contributions have been made to develop contextually appropriate, anti-racist approaches to mental health care by people including Fanon, Lambo, Hickling, Burke, and Fernando. This literature has generally not been integrated into mainstream histories of psychiatry, and offers a rich resource for the contemporary field of mental health research. Indeed, the misogynoir that has ‘hidden figures’ and historically shut Black women out of psychiatry or relegated them to the margins of the psy-disciplines, literature, and citations, has meant that this article has only made a start in charting the actors who have challenged racist aetiologies. Moreover, the ongoing lack of investment in overseas scholars and the current loss of funding to the history of medicine in so-called ‘developed nations’ may mean that there will be limited decolonisation, diversification and other critical unpacking in the fields of health and science at a time when we need it most.
